# Editorial: Use of big data and artificial intelligence in multiple sclerosis

**DOI:** 10.3389/fimmu.2025.1679482

**Published:** 2025-08-20

**Authors:** Liesbet M. Peeters, Axel Faes, Hans-Peter Hartung

**Affiliations:** ^1^ University MS Center (UMSC), Hasselt-Pelt, Belgium; ^2^ Biomedical Research Center (BIOMED), Hasselt University, Diepenbeek, Belgium; ^3^ Data Science Institute (DSI), Hasselt University, Diepenbeek, Belgium; ^4^ Department of Neurology, Heinrich-Heine-University Düsseldorf, Düsseldorf, Germany; ^5^ Brain and Mind Center, University of Sydney, Sydney, NSW, Australia; ^6^ Department of Neurology, Palacky University Olomouc, Olomouc, Czechia

**Keywords:** multiple sclerosis (MS), artificial intelligence, clinical decision support, magnetic resonance imaging, digital health monitoring, biomarkers, trustworthy machine learning

## Introduction

As health data volume and the sophistication of artificial intelligence (AI) tools grow, their potential to transform the management of complex neuroimmunological conditions like multiple sclerosis (MS) has become increasingly evident (1). MS, a chronic immune-mediated inflammatory disorder of the central nervous system, presents a unique challenge in the health sector due to its multifactorial nature and variable progression patterns. Each patient’s journey is marked by distinct symptom trajectories and responses to treatment, demanding personalised approaches in diagnosis, prognosis, and therapeutic interventions. (2, 3).

This Special Topic aims to address the clinical complexity of MS by leveraging data driven insights and innovative health initiatives. The overarching goal is to present the current challenges in MS research and explore recent advances and future trends that can significantly impact patient care. Through a Research Topic of reviews, perspectives, and original research articles, we explore how advanced data techniques and innovative health initiatives are shaping the future of MS research and care.

## Inspiring examples to showcase the potential

We kick-start with spotlighting a recently approved European Project, ‘Clinical Impact through AI-assisted MS Care’ (CLAIMS). Praet et al. explains how this project will develop, validate and seek regulatory approval for an AI-driven clinical decision-support platform, which offers the MS care team a holistic view of the patient through the visualisation of all relevant patient data and the prognosis on the expected disease trajectories under different treatment regimens. Next to this, two original research contributions further illustrate AI’s capacity to enhance MS treatment personalization and early diagnosis. Ilan et al. examine how advanced AI systems can help personalise and diversify treatment regimens, reducing the risk of drug tolerance. Meanwhile, Albuz et al. examine how AI-assessed volumetric measurements of specific brain regions correlate with neuropsychological test outcomes in patients with clinically isolated syndrome, illuminating potential early indicators of MS.

## Magnetic resonance imaging and AI in MS

MRI remains central to diagnosing, monitoring, and optimising MS treatment due to its ability to non-invasively visualise both lesional and nonlesional brain pathology. However, the potential of MRI is often constrained in clinical practice by lengthy protocols, challenges in lesion identification, and limited predictive power regarding disability progression. Falet et al. highlight recent AI advances that could enhance MRI’s accuracy and broaden its predictive capabilities, improving critical patient outcomes.

## Digital tools and AI in MS

The integration of digital monitoring tools, big data, and AI presents new possibilities for real-time tracking of MS symptoms and progression. Dini et al. explore the latest advancements in digital remote monitoring, with devices like wearables and smartphones playing an increasing role in the field. These technologies, coupled with AI analytics, are demonstrating reliability in assessing motor symptoms such as fall risk and gait irregularities, both in clinical settings and through passive, real-life monitoring. While cognitive monitoring is still evolving, AI-driven tools are now beginning to automate neuropsychological test scoring and passive keystroke analysis, setting the stage for continuous, long-term data collection on both motor and cognitive symptoms.

## Biomarkers and AI in MS

Expanding the scope to biological markers, Arrambide et al. delve into AI methodologies applied to serum, blood, and cerebrospinal fluid (CSF) biomarkers, outlining key studies, limitations, and future directions. Notably, this systematic review reveals that most research papers on AI applications to biomarker data in MS have been published within the past four years, underscoring that this field is still in its early stages and remains some distance from widespread clinical application.

## Future trends

Recognizing the necessity of reliable and interpretable machine learning (ML) in MS, Werthen-Brabants et al. emphasise the need for Trustworthy ML. Given the complex and individualised nature of MS, these authors advocate collaborative efforts among researchers, clinicians, and policymakers to develop ML solutions that are technically robust, clinically relevant, and patient-centred.

Patient-reported outcome measures (PROMs) are vital for capturing the lived experiences of people with MS, providing insights that enrich clinical understanding. However, PROMs are underutilised in both clinical research and routine care. Helme et al. discuss the challenges in scaling PROMs and highlight efforts to integrate health outcomes data across Europe and beyond, noting initiatives like the European Health Data Space (EDHS) that may expand their application.

While the MS community has made substantial progress in leveraging data for research and patient care, several large-scale collaborative efforts across Europe—though not exclusively focused on MS—have the potential to transform the management and application of health data across various diseases, including MS. Peeters highlights key initiatives such as the EHDS, DARWIN-EU, the Observational Health Data Sciences and Informatics (OHDSI), EBRAINS, and ELIXIR. She outlines the challenges that remain in aligning with these initiatives and offers concrete, actionable recommendations to guide the MS research community toward more effective integration and collaboration.

## Conclusion

We believe this special topic has opened new perspectives, and gives us some indications of where the field of Big Data and AI in MS is heading. First of all, it testifies that the domain is expanding rapidly. At the same time, however, researchers will have to solve some open issues, such as the need to develop trustworthy, reliable AI models, consistently capture multidimensional longitudinal data, incorporate the patient perspectives and the alignment with evolving regulatory frameworks such as the EHDS. We hope you find this Research Topic as inspiring and impactful to read as it was for us to prepare.

## In memoriam

In memoriam of Prof. Giancarlo Comi who helped to launch this project and like many other areas fertilized and promoted the field.
